# CHAC1 as a Novel Contributor of Ferroptosis in Retinal Pigment Epithelial Cells with Oxidative Damage

**DOI:** 10.3390/ijms24021582

**Published:** 2023-01-13

**Authors:** Ye Liu, Di Wu, Qiuli Fu, Shengjie Hao, Yuzhou Gu, Wei Zhao, Shuying Chen, Feiyin Sheng, Yili Xu, Zhiqing Chen, Ke Yao

**Affiliations:** Zhejiang Provincial Key Lab of Ophthalmology, Eye Center of the Second Affiliated Hospital, School of Medicine, Zhejiang University, Hangzhou 310009, China

**Keywords:** *Chac1*, retinal pigment epithelial (RPE), oxidative stress, ferroptosis, age-related macular degeneration (AMD)

## Abstract

Age-related macular degeneration (AMD) is the leading cause of irreversible visual loss in the elderly population. With aging and the accumulated effects of environmental stress, retinal pigment epithelial (RPE) cells are particularly susceptible to oxidative damage, which can lead to retinal degeneration. However, the underlying molecular mechanisms of how RPE responds and progresses under oxidative damage are still largely unknown. Here, we reveal that exogenous oxidative stress led to ferroptosis characterized by Fe^2+^ accumulation and lipid peroxidation in RPE cells. Glutathione specific gamma-glutamylcyclotransferase 1 (*Chac1*), as a component of the unfolded protein response (UPR) pathway, plays a pivotal role in oxidative-stress-induced cell ferroptosis via the regulation of glutathione depletion. These results indicate the biological significance of *Chac1* as a novel contributor of oxidative-stress-induced ferroptosis in RPE, suggesting its potential role in AMD.

## 1. Introduction

Age-related macular degeneration (AMD) is the leading cause of irreversible blindness in the elderly worldwide [1]. Globally, it is estimated that 196 million people suffered from this disease in 2020; as the population grows, this number might grow to 288 million by 2040 [2]. Clinically, AMD is classified into the non-neovascular or atrophic form (known as “dry AMD”) and the neovascular or exudative form (known as “wet AMD”). More than 80% of AMD patients have dry AMD; however, this type may progress to wet AMD, which is characterized by choroidal neovascularization (CNV), which extends from the choroid into the subretinal space and disrupts Bruch’s membrane and the retinal pigment epithelium (RPE), causing severe vision loss [3]. Currently, there are several treatment options available for wet AMD using anti-angiogenic agents, but no effective therapy has been developed for dry AMD.

The RPE plays a critical role in maintaining normal vision due to its anatomic location between the choriocapillaris and light-sensitive outer segments of the photoreceptors, and its specific functions, e.g., the daily phagocytosis of photoreceptor outer segments, the secretion of the neurotrophic factors required to stabilize the neural retina and scavenging for damaging reactive oxygen species (ROS) [4]. With aging, the accumulation of ROS leads to an imbalance in the homeostatic conditions of the retina and subsequent oxidative damage in RPE cells vulnerable to oxidative injury. A number of studies indicated that constant oxidative damage could activate inflammatory reactions [5], autophagy dysregulation [6], cellular senescence [7], and programmed cell death (PCD) [8], all of which are involved in the pathological phenotype of AMD.

Glutathione specific gamma-glutamylcyclotransferase 1 (*Chac1*), a candidate gene for the glutathione (GSH)-metabolism signaling pathway, has been reported to have higher catalytic efficiency and hydrolyzed glutathione in the cytosol of mammalian cells via its γ-glutamylcyclotransferase activity to 5-oxoproline and cysteinyl glycine [9]. Considering the biological role of glutathione in regulating intracellular antioxidants [10] and the progression of cell death [11], several studies reported that *Chac1* mediated cellular fate via glutathione metabolism pathways [12,13,14]. However, the molecular mechanism underlying *Chac1* in AMD remains unknown.

In the present study, we revealed for the first time that *Chac1* could be a significant contributor to ferroptosis but not apoptosis in RPE, which is possibly related to the ER-stress response to oxidative damage.

## 2. Results

### 2.1. Oxidative Stress Is Involved in Laser-Induced CNV Mice Model

**Figure 1:**
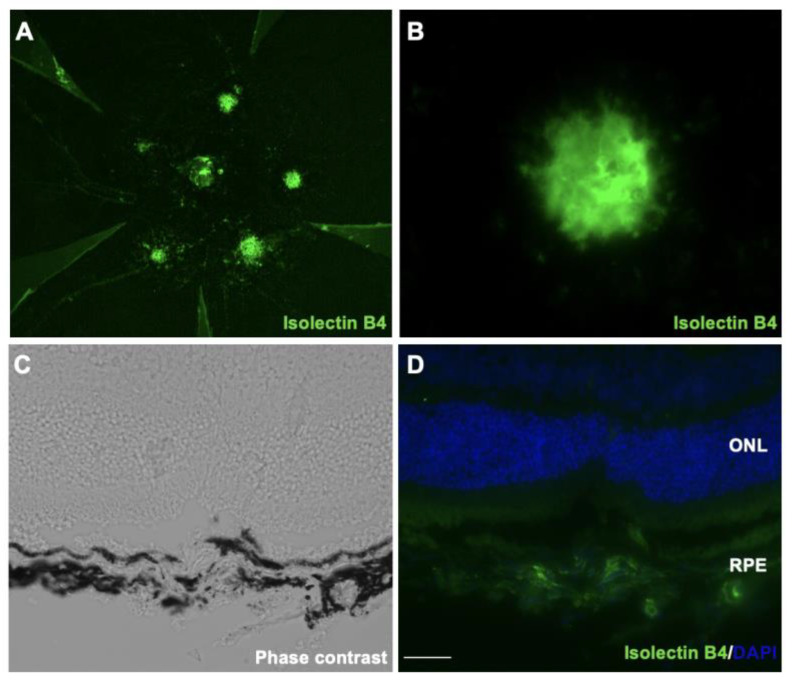

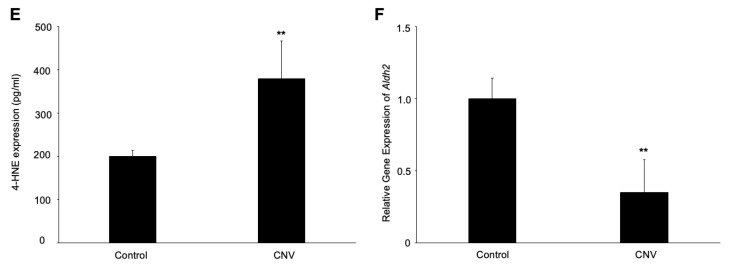
Immunofluorescence images of the isolectin B4-stained RPE/choroidal flat-mount of laser-induced CNV mice. (**A**,**B**) Labeling of isolectin B4 (green) in eyes with CNV from mice at post-laser day 7. (**C**) Phase-contrast image of CNV section. (**D**) Immunofluorescence image of CNV section. Scale bar, 20 μm. (**E**) 4-HNE expression levels in RPE/choroidal complex at post-laser day 7. (**F**) *Aldh2* gene expression levels in RPE/choroidal complex at post-laser day 7. *n* = 6 in each group. ** *p <* 0.01.

Laser-induced CNV, which is widely used as an animal model for AMD, reflects at least partially the pathogenesis of human neovascular AMD. To check whether oxidative stress is involved in the pathogenesis of CNV, we first established a laser-induced CNV model. Next, we found that the isolectin B4 stained new vessels penetrating the subretinal space, and the melanin in RPE/choroidal complex was missing at post-laser day 7 (Figure 1A–D). After laser injury, we then examined the protein-expression level of 4-HNE, an oxidative stress marker. As shown in Figure 1E, the 4-HNE protein levels in the RPE/choroid complex of the CNV mice significantly increased to 1.9 times (*p* < 0.01) compared with the untreated mice. Additionally, we also found that the gene expression levels of aldehyde dehydrogenase 2 (*Aldh2*), a mitochondrial enzyme that metabolizes 4-HNE [15], was significantly decreased at post-laser day 7 (Figure 1F). These results indicated the involvement of oxidative stress in the pathogenesis of CNV.

### 2.2. Hydrogen Peroxide Suppressed RPE-Cell Viability Not via Apoptosis

**Figure 2:**
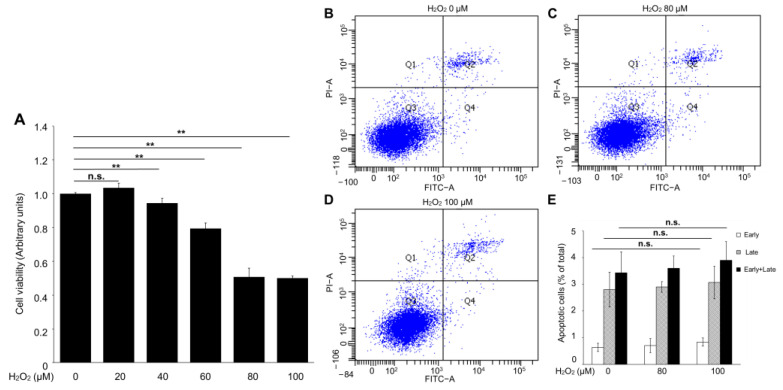
The apoptotic effect of hydrogen peroxide on ARPE-19 cells. (**A**) Cell-viability assay. (**B**–**E**) Representative flow-cytometer dot plot of apoptosis in ARPE-19 cells. *n* = 6 in each group, n.s. denotes not significant. ** *p* < 0.01.

Hydrogen peroxide, the primary source of ROS, is widely used as a pre-clinical model of RPE-cell dystrophy [16]. To conduct an in vitro experiment to mimic an in vivo study, RPE cells were treated with various concentrations of hydrogen peroxide for 24 h, and a CCK-8 assay was performed. As shown in Figure 2A, the hydrogen-peroxide administration led to a significant decrease in cell viability in a dose-dependent manner, and the IC_50_ (the half maximal inhibitory concentration) of the hydrogen-peroxide-treated ARPE-19 cells was 80 to 100 μM.

To investigate whether the hydrogen-peroxide-induced cell-viability loss was mediated by apoptosis in ARPE-19 cells, an Annexin V-FITC binding assay was performed to evaluate the percentage of early apoptosis (Annexin V-FITC+/PI−) and late apoptosis (Annexin V-FITC+/PI+). Interestingly, no significant changes were observed in the early and late apoptosis of the ARPE-19 cells with or without hydrogen peroxide administration (Figure 2B–E). These results suggested that apoptosis is not the major mediator of hydrogen-peroxide-induced ARPE-19 cell death.

### 2.3. Hydrogen-Peroxide-Induced Ferroptosis in RPE Cells

**Figure 3:**
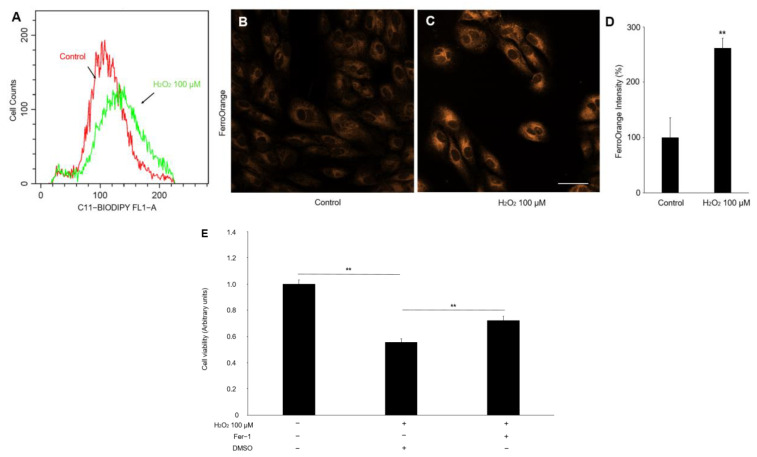
The lipid peroxidation and Fe^2+^ level in the ARPE-19 cells treated with hydrogen peroxide. (**A**) The fluorescence histogram plot of the lipid-peroxidation production in ARPE-19 cells by BODIPY^TM^ 581/591. (**B**–**D**) Representative fluorescent images and quantitative analysis of intracellular iron level in ARPE-19 cells using FerroOrange (orange). Scale bar, 50 μm. (**E**) Cell viability assay. *n* = 5 in each group, ** *p* < 0.01.

Since ferroptosis cell death is closely related to oxidative stress, we next assessed the effect of hydrogen peroxide on lipid peroxidation and Fe^2+^ concentration in the ARPE-19 cells, which are major hallmarks of ferroptosis [17]. As shown in Figure 3A, the C11 BOPDIPY^581/591^ fluorescence assays demonstrated a marked increase in the lipid peroxidation of the APRE-19 cells treated with hydrogen peroxide. Similarly, the FerroOrange staining also found a significant increase in fluorescence intensity, indicating that the hydrogen peroxide administration upregulated the Fe^2+^ concentration in the ARPE-19 cells (Figure 3B–D). Moreover, we pretreated Fer-1(8 μM), a selective inhibitor of ferroptosis, before hydrogen peroxide treatment and found an increase in RPE-cell viability (Figure 3E). These data suggested that ferroptosis is a crucial pathological process in oxidative-stress-induced RPE damage.

### 2.4. Hydrogen Peroxide Upregulated Chac1 Production in ARPE-19 Cells

**Figure 4:**
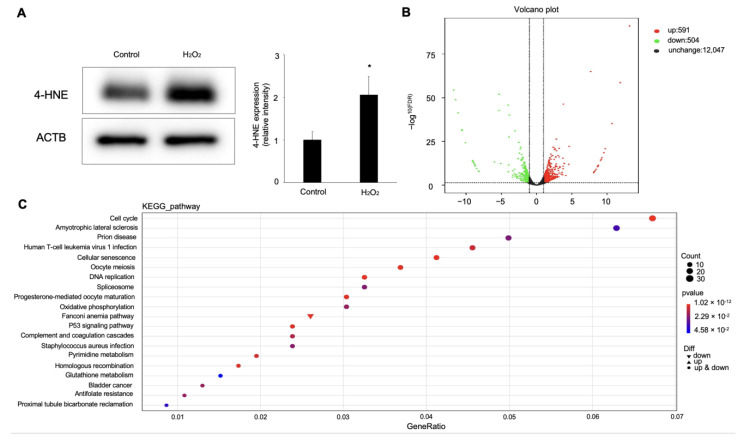
Differential-expression analysis of ARPE-19 cells with hydrogen peroxide stimulation. (**A**) Western blot analysis for 4-HNE in ARPE-19 cells treated with 100 μM hydrogen peroxide. *n* = 3 in each group. * *p* < 0.05. (**B**) Visualization of RNA-Seq results with Volcano plot. (**C**) KEGG-pathway classification for differentially expressed genes.

To investigate the underlying molecular mechanism of hydrogen-peroxide-induced ferroptosis in RPE cells, we conducted RNA-Seq and bioinformatics analyses to check the gene-expression levels in the hydrogen-peroxide-treated RPE cells, in which 4-HNE expression was upregulated (Figure 4A). As a result, 591 upregulated (fold change ≥ 2) and 504 downregulated (fold change ≤ 0.5) genes were identified in the hydrogen-peroxide-treated group compared to the control (Figure 4B). To verify the reliability of the gene-expression data obtained by the RNA-Seq, four genes (*Ftl*, *Mfn1*, *Mfn2*, and *Fth1*) were randomly selected for qRT-PCR detection. For each gene, the qRT-PCR expression results showed a similar tendency to the RNA-Seq results, suggesting the RNA-Seq results were credible in this study. To investigate the involved pathway of the DEGs, we performed KEGG-pathway annotation. The KEGG analysis showed that 20 KEGG pathways were significantly enriched for these DEGs, which are mainly associated with cellular processes, metabolism, and genetic information processing (Figure 4C).

Based on the results of the KEGG-pathway analysis, the GSH metabolism pathway, as one of the most obviously changed pathways, drew our attention. Hence, we next focused on the genes enriched in the GSH-metabolism pathway, shown in Table 1. Among these, *Chac1* was the most highly upregulated gene in the ARPE-19 cells stimulated with hydrogen peroxide.

**Figure 5:**
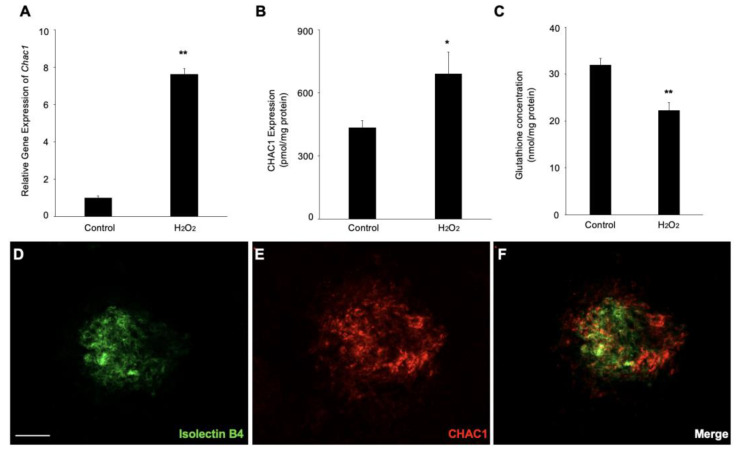
Gene and protein expression levels of *Chac1* in ARPE-19 cells with hydrogen-peroxide stimulation. (**A**) Real-time PCR analysis for *Chac1*. (**B**) CHAC1 protein levels in ARPE-19 cells with hydrogen peroxide stimulation. (**C**) GSH assay. (**D**–**F**) Double labeling of CHAC1 (red) and isolectin B4 (green) in CNV mice at day 7. Scale bar = 100 μm. *n* = 6 in each group. * *p* < 0.05, ** *p* < 0.01.

The real-time PCR and ELISA were performed to confirm further that the *Chac1* mRNA expression and protein production were significantly increased by the hydrogen-peroxide treatment (Figure 5A,B). Meanwhile, we examined the concentration of GSH under these treatments to validate the imbalance in glutathione homeostasis. As expected, the concentration of GSH was significantly decreased in the RPE with the hydrogen peroxide (Figure 5C), which demonstrated that hydrogen peroxide upregulates *Chac1* and reduces GSH production in RPE cells. Furthermore, to investigate whether *Chac1* is involved in the pathogenesis of CNV, we performed immunostaining to examine the localization of CHAC1 in the flat-mounts of RPE/choroid complexes in CNV mice. Immunostaining analyses showed that CHAC1 was localized in the CNV area stained by isolectin B4 on day seven after laser injury (Figure 5D–F). Taken together, our data suggest that *Chac1*, a candidate gene for the GSH metabolism pathway, is involved in the pathogenesis of oxidative stress damage in RPE cells as well as laser-induced CNV in mice.

### 2.5. CHAC1 Mediated GSH Metabolism in RPE Cells Treated with Hydrogen Peroxide

**Figure 6:**
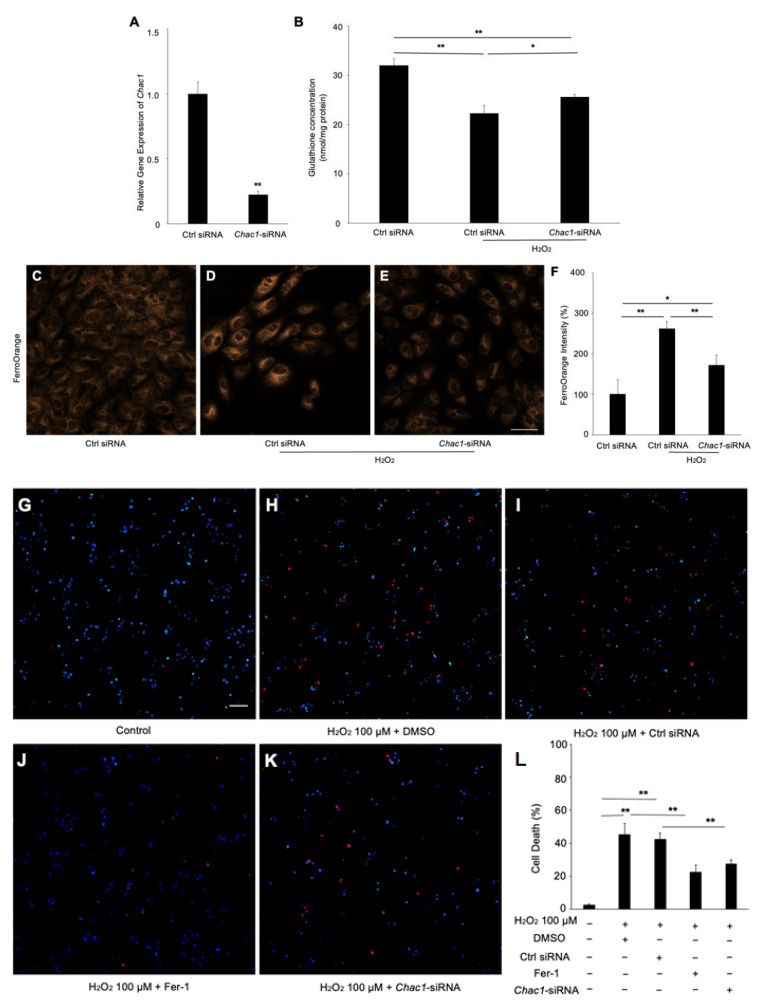
The effect of *Chac1* silencing on ferroptosis and cell death of RPE cells treated with hydrogen peroxide. (**A**) Gene silencing effect of *Chac1* siRNA in ARPE-19 cells. (**B**) GSH assay. (**C**–**F**) Representative fluorescent images and quantitative analysis of intracellular iron level in ARPE-19 cells using FerroOrange (orange). (**G**–**L**) Cell death analysis using PI staining. Scale bar, 50 μm. *n* = 5 in each group. * *p* < 0.05, ** *p* < 0.01.

To investigate whether *Chac1* contributes to ferroptosis through GSH depletion in RPE cells subject to oxidative damage, we first checked the gene silencing efficiency of *Chac1*-siRNA in ARPE-19 cells (Figure 6A). We then examined the effect of *Chac1* silencing on GSH concentration and intracellular iron level in RPE cells with hydrogen peroxide treatment. As shown in Figure 6B, the concentration of GSH was significantly increased by the *Chac1* siRNA compared with the control siRNA. In addition, the *Chac1* siRNA markedly reduced the excessive Fe^2+^ in the hydrogen-peroxide-treated RPE cells (Figure 6C–F). Furthermore, propidium iodide (PI) staining was performed to evaluate the effect of *Chac1* silencing on hydrogen-peroxide-induced RPE cell death (Figure 6G–L). As shown in Figure 6H,I, hydrogen peroxide treatment significantly increased cell death in ARPE-19 cells, which could be rescued by Fer-1 or *Chac1*-siRNA treatment (Figure 6J,K). These data indicated that *Chac1* contributes to ferroptosis by regulation of GSH metabolism in RPE cells under oxidative damage.

## 3. Discussion

In this study, we obtained the following novel findings: (1) hydrogen peroxide induces dysfunction in cell viability of ARPE-19 cells, predominantly via ferroptosis and not through apoptosis; (2) 1095 genes were changed with hydrogen peroxide treatment, among which, *Chac1* was most highly upregulated; (3) *Chac1* was involved in the pathogenesis of laser-induced CNV in mice; (4) *Chac1* could facilitate ferroptosis via accelerated GSH deprivation in RPE cells. Altogether, our study emphasizes the critical role of oxidative damage to RPE cells in AMD and provides evidence that hydrogen peroxide induces GSH depletion via *Chac1* signaling, which leads to ferroptosis in RPE cells.

Aging, environmental influence, and genetic susceptibility have been demonstrated to contribute to the pathogenesis of AMD by inducing oxidative damage [18]. Additionally, several well-known oxidative stress markers, such as malondialdehyde (MDA) and advanced glycation end-products (AGE), are highly expressed in AMD patients, further suggesting that oxidative stress is an important factor in the mechanism of AMD development [19,20]. While vision loss is due to damage to photoreceptors in AMD patients, RPE cell damage and death are also involved [21]. Here, we constructed a laser-induced CNV mouse model and found that 4-HNE was significantly increased in the RPE/choroidal complexes of mice with CNV at post-laser day 7, which is consistent with the previous finding that oxidative damage facilitates the degeneration of RPE cells and participates in the pathogenesis of AMD. Furthermore, the inactivation of *Aldh2* might contribute to elevated oxidative stress, which leads to a reduction of reactive aldehyde metabolism and 4-HNE accumulation.

The exogenous hydrogen-peroxide-induced accumulation of intracellular ROS through the simulation of endogenous ROS signaling pathways led to cell viability suppression and triggering PCD [22]. Recently, in vitro and in vivo studies have demonstrated that forms of PCD, such as apoptosis, pyroptosis, necrosis, and ferroptosis, contribute to RPE cell death and the pathogenesis of AMD [20]. Ferroptosis, a new form of PCD, was characterized by lipid peroxidation and glutathione depletion mediated by iron metabolism [23]. Concerning retinal diseases, accumulated iron has been found in the RPE, photoreceptors, and Bruch’s membrane in AMD patients [24,25]. Meanwhile, the expression of iron-metabolic genes, such as transferrin (TF) and ferritin light chain (FTL) has also been reported to be significantly increased in the aged retina [26]. Previously, iron-metabolism inhibition was found to be protective against AMD [27,28]. Our results show that hydrogen-peroxide-induced lipid peroxidation and iron accumulation, which supports the recent finding that ferroptosis plays an important role in oxidative-stress-induced RPE cell death [29]. Generally, ferroptosis involves genetic changes in iron homeostasis [30], lipid-peroxidation metabolism [31], and the glutathione metabolic pathway [32], but the molecular mechanisms underlying the interplay between oxidative stress and ferroptosis in RPE cells are still largely unknown.

As a component of the unfolded protein response (UPR) pathway, *Chac1* is known to be a candidate gene for the glutathione-metabolism-signaling pathway, which accelerates glutathione degradation. Considering that glutathione is a crucial intracellular antioxidant, several studies showed that the dysregulation of *Chac1* alters intracellular glutathione expression and is associated with various diseases, such as cancer [33] and heart failure [34]. Meanwhile, the silencing of *Chac1* has recently been found to ameliorate heat stress (HS)-induced intestinal porcine epithelial cell apoptosis due to the upregulation of glutathione, thereby relieving HS-related intestine impairment [35]. In the context of ocular diseases, glutathione depletion induced ferroptosis [36], apoptosis [37], and necrosis [38], which has been found to be involved in the development of ocular diseases, especially in AMD. To the best of our knowledge, the present study demonstrated for the first time the upregulation of *Chac1* in oxidative damaged RPE cells, while the silencing of *Chac1* significantly ameliorated ferroptosis via the inhibition of glutathione depletion. The aggregation of misfolded and unfolded proteins is considered the excess production in the lumen following an imbalance in the oxidative environment of cells and leads to endoplasmic reticulum (ER) stress [39]. This complex situation activates the UPR and leads to the activation of downstream signaling pathways, such as inositol-requiring protein-1 (IRE1), activating transcription factor-6 (ATF6) or protein kinase R-like endoplasmic reticulum kinase (PERK). It was shown that *Chac1* activity was associated with ER-stress response under external stress [40]. In our RNA-seq analysis, CCAT/enhancer-binding protein homologous protein (CHOP), a molecular target of the PERK-elF2alpha-ATF4 branch of the UPR/ER-stress pathway, was found to be upregulated 2.1 times (*p* < 0.05) in hydrogen-peroxide-induced RPE cells by using RNA-seq analysis. These results imply that the ER-stress-signaling pathway could be a trigger for the transcription of *Chac1* and that it could contribute to ferroptosis by degrading intracellular glutathione in RPE cells subject to oxidative damage. Future studies are needed to clarify whether *ChaC1* itself could induce or promote ferroptosis in RPE cells.

In conclusion, our results demonstrate, for the first time, that *Chac1* is a novel contributor to ferroptosis in RPE cells with oxidative damage, suggesting its potential role in the pathogenies of AMD.

## 4. Materials and Methods

### 4.1. Animals

C57BL/6J mice aged 6–8 weeks were purchased (Charles River Lab China, Hangzhou, China) and maintained in the Animal Facility at Zhejiang University. All animal experiments were performed following the guidelines of the Association for Research in Vision and Ophthalmology (ARVO) Statement for the Use of Animals in Ophthalmic and Vision Research. The experiments were approved by the Institutional Animal Care and Use Committee at Zhejiang University.

### 4.2. Laser-Induced CNV Model

CNVs were generated as described previously [41,42]. In brief, male mice (6–8 weeks old) were anesthetized, and pupils were dilated with 0.5% phenylephrine and 0.5% tropicamide. CNV was induced with 532-nanometer laser photocoagulation (180 mW, 50 μm, 100 ms, VISULAS; Carl Zeiss, Jena, Germany). Four laser spots were performed around the optic disc using a slit-lamp delivery system with a cover glass as a contact lens. Disruption of Bruch’s membrane was confirmed by the formation of a subretinal bubble at the time of laser injury.

### 4.3. Assessment of CNV

Seven days after laser treatment, the eyes were enucleated and fixed in 4% paraformaldehyde. The anterior segment and the retina were removed from the eyeball. Five radial relaxing incisions were made, and the remaining RPE-choroid complex was stained with Isolectin B4-Alexa 488 (1:100; Thermo Fisher Scientific, Waltham, MA, USA) to detect CNV and incubated with rabbit anti-CHAC1 antibody (Proteintech Group, Rosemon, IL, USA). The secondary antibody for fluorescent detection was Alexa Fluor 555 (1:100; Thermo Fisher Scientific). A Leica DM6B fluorescence microscope (Leica, Wetzlar, Hesse, Germany) was used to observe the CNV.

### 4.4. Enzyme-Linked Immunosorbent Assay (ELISA)

Total protein was isolated from the sonicated mouse RPE/choroid complexes using pre-cooling PBS. The expression levels of 4-HNE protein in the murine RPE/choroid complexes were measured using the mouse 4-HNE (4-hydroxynonenal) ELISA kit (MyBiosource, San Diego, CA, USA), according to the manufacturer’s instructions. The concentration of 4-HNE was normalized by the total protein concentration of the RPE/choroid complexes measured using the bicinchoninic-acid Protein Assay Kit (BCA; Thermo Fisher Scientific).

### 4.5. Cell Culture

The hRPE cell line, ARPE-19 (ATCC, Manassas, VA, USA), was cultured in Dulbecco’s modified Eagle’s medium (DMEM; Gibco, Gaithersburg, MD, USA)/F12 (1:1) containing 10% fetal bovine serum at 37 °C. The following experiments were performed at a 5% CO_2_ and a 20% O_2_ atmosphere. All experiments were performed with cells in a subconfluent state at passages 15 to 30.

### 4.6. Cell Viability Assay

The ARPE-19 cells were seeded into a 96-well plate and treated with concentrations of hydrogen peroxide varying between 10 μM and 100 μM for 24 h. The cell viability was assessed with the Cell Counting Kit-8 (CCK-8; DojinDo, Kumamoto, Japan) for absorption measurements at 450 nm using a microplate reader (Bio-Rad, Hercules, CA, USA). Experiments were conducted in six parallel wells and repeated at least three times.

### 4.7. Intracellular Glutathione Assay

The intracellular levels of glutathione were measured using a standard GSH and GSSG assay kit (Beyotime, Shanghai, China). Briefly, ARPE-19 cells were seeded into a 6-well plate at a density of 3 × 10^5^ cells per well and treated with or without 100 μM hydrogen peroxide for 24 h. Cells were harvested and re-suspended in 10 μL of cell medium and were counted using a hemocytometer. Twenty microliters of cells were mixed with 60 μL 5% metaphosphoric acid, after which the cells were frozen and thawed twice using liquid nitrogen and 37 °C water. Next, the samples were centrifuged and the supernatant was used for GSH assay. The intracellular levels of GSH were measured by absorption measurements at 412 nm using a microplate reader. Three independent experiments were conducted.

### 4.8. Lipid-Peroxidation Detection

Lipid peroxidation was examined by C11-BODIPY^581/591^ (Invitrogen, Carlsbad, CA, USA) according to the manufacturer’s protocol. In brief, ARPE-19 cells were incubated with C11-BODIPY^581/591^ at a final concentration of 10 μM for 30 min at 37 °C and washed three times with PBS. The fluorescence shifted from red to green according to the cell state (non-peroxidized: red; peroxidized: green). Flow cytometric analysis detected and measured these changes (FACScan; BD, Piscataway, NJ, USA).

### 4.9. Iron Assay

The iron concentration was assessed using FerroOrange (DojinDo, Kumamoto, Japan), according to the manufacturer’s protocol. ARPE-19 cells were treated with hydrogen peroxide for 24 h and stained with a final concentration of 1 μmol/L FerroOrange at 37 °C. The cells were incubated for 30 min and observed under a Leica TCS SP8 confocal microscope (Leica, Wetzlar, Germany). Fluorescence intensities were calculated using ImageJ software (National Institutes of Health, Washington, DC, USA). Three independent experiments were conducted.

### 4.10. RNA-Seq Analysis

Total RNA of each sample was extracted with TRlzol Reagent (Life technologies, Carlsbad, CA, USA). The purity, concentration, and integrity of the total RNA samples were assessed before further analysis. The RNA-seq libraries were constructed using Illumina TruSeq RNA sample kit (Illumina, San Diego, CA, USA). DESeq2 performed gene-expression analysis of the different groups. Genes with a *p*-value of <0.05 were defined as differentially expressed genes (DEGs).

### 4.11. Real-Time Quantitative-Polymerase Chain Reaction (qPCR)

Following the manufacturers’ instructions, total RNA was isolated using TRIzol Reagent, and reverse transcription was then performed to cDNA with PrimeScript^TM^ RT Master Mix (Takara, Beijing, China). The primer sequences used for real-time qPCR were 5′-TTGCCTCCCATGAGGATGTGGA-3′ (forward) and 5′-GGTCACTCTCTTGAGGTT GCT G-3′ (reverse) for *Aldh2*, 5′-GAACCCTGGTTACCTGGGC-3′ (forward) and 5′-CGC AGCAAGTATTCAAGGTTGT-3′ (reverse) for *Chac1*, and 5′-GGGAAATCGTGCGTG ACATT-3′ (forward) and 5′-GCGGCAGTGGCCATCTC-3′ (reverse) for *Actb*. Real-time qPCR was performed using the SYBR Premix Ex Tag^TM^ (Takara, Beijing, China) with ABI Fast 7500 RT-PCR system (Life Technologies, v2.0.6). Gene-expression levels were calculated using the 2-^dd^CT method, and all experimental samples were normalized using Actb as the internal control.

### 4.12. ELISA for CHAC1

ARPE-19 cells were treated with or without hydrogen peroxide for 24 h. Levels of CHAC1 protein in the cell lysate were analyzed using ELISA kits for human CHAC1 (MyBioSource, San Diego, CA, USA) following the manufacturer’s protocol. Absorbance was read at 450 nm on a microplate reader (Bio-Rad, Hercules, CA, USA), and CHAC1 concentration was normalized by the total protein concentration of cell lysates measured by BCA kit.

### 4.13. RNA Interference

ARPE-19 cells were transfected with siRNA to silencing the gene expression of *Chac1* (*Chac1*-siRNA; Genechem, Shanghai, China) and negative control (Ctrl-siRNA). Transfections were performed using the lipofectamine RNAiMAX reagent (Thermo Fisher Scientific). The composite transfection mixture was replaced with 10%FBS/DMEM 24 h after the transfection.

### 4.14. Statistical Analysis

Data were expressed as the mean ± standard error of the mean from at least three biological replicates. The differences between the two groups were analyzed by Student’s t-test. For comparisons of multiple groups, one-way ANOVA analysis and Tukey’s honestly significant difference multiple-comparisons test were used. A value of *p* < 0.05 was considered statistically significant.

## Figures and Tables

**Table 1:** Three genes upregulated by hydrogen peroxide in ARPE-19 cells.

No.	Gene Symbol	GeneBank Accession	Full Name	Ratio of Control
1	*Chac1*	NM_001142776	ChaC glutathione specific gamma-glutamylcyclotransferase 1	5.6
2	*Ggt1*	NM_001288833	gamma-glutamylcyclotransferase 1	3.2
3	*G6pd*	NM_000402	Glucose-6-phosphate dehydrogenase	2.0

## Data Availability

The datasets analyzed during the current study are available from the corresponding author upon reasonable request.
